# The Associations of Novel Vitamin D_3_ Metabolic Gene *CYP27A1* Polymorphism, Adiponectin/Leptin Ratio, and Metabolic Syndrome in Middle-Aged Taiwanese Males

**DOI:** 10.1155/2015/658151

**Published:** 2015-01-05

**Authors:** Kai-Hung Cheng, Edward Hsi, Chia-Chu Liu, Chun-Nung Huang, Yung-Chin Lee, Chih-Sheng Chu, Bo-Ying Bao, Chu-Fen Chang, Shu-Pin Huang, Po-Lin Kuo, Wen-Ter Lai

**Affiliations:** ^1^Division of Cardiology, Department of Internal Medicine, Kaohsiung Medical University Hospital, 100 Tzyou 1st Road, Kaohsiung 80708, Taiwan; ^2^Faculty of Medicine, College of Medicine, Kaohsiung Medical University, Kaohsiung 80708, Taiwan; ^3^Graduate Institute of Medicine, Kaohsiung Medical University, Kaohsiung 80708, Taiwan; ^4^Department of Medical Research, Kaohsiung Medical University Hospital, Kaohsiung 80708, Taiwan; ^5^Department of Urology, Kaohsiung Medical University Hospital, 100 Tzyou 1st Road, Kaohsiung 80708, Taiwan; ^6^Department of Urology, College of Medicine, Kaohsiung Medical University, Kaohsiung 80708, Taiwan; ^7^Pingtung Hospital, Department of Health, Executive Yuan, Pingtung 90054, Taiwan; ^8^Department of Pharmacy, China Medical University, Taichung 40402, Taiwan; ^9^Department of Physical Therapy, Tzu Chi University, Hualien 97004, Taiwan; ^10^Institute of Clinical Medicine, College of Medicine, Kaohsiung Medical University, Kaohsiung 80708, Taiwan

## Abstract

Metabolic syndrome (MetS) confers increased risks of cardiovascular disease (CVD). Both vitamin D_3_ and adipocytokines (especially adiponectin and leptin) have a great impact on CVD and MetS. In vitamin D_3_ metabolism, the vitamin D_3_ 25-hydroxylase (CYP27A1) and 25-hydroxyvitamin D_3_ 1-alpha-hydroxylase (CYP27B1) are two key enzymes. This study aimed to examine the influence of vitamin D_3_ CYP27 single nucleotide polymorphisms (SNPs) on adipocytokines and MetS. Cross-sectional data and DNA samples were collected from male volunteers (*n* = 649, age: 55.7 ± 4.7 years). Two tagging SNPs, *CYP27A1 rs4674344* and *CYP27B1 rs10877012*, were selected from the HapMap project. MetS was significantly associated with the *CYP27A1 rs4674344* SNP (*P* = 0.04) and the ratio of adiponectin/leptin (A/L ratio) was most correlated to the *CYP27A1 rs4674344* SNP, appearing to be significantly lower in T-carriers than in AA subjects (3.7 ± 4.0 versus 5.1 ± 6.0, *P* = 0.001) and significantly negatively associated after adjustment. For each MetS component associated with the *CYP27A1 rs4674344* SNP, the A/L ratios were significantly negative in preclinical stage (condition not meeting the individual criteria), except the blood pressure. In conclusion, *CYP27A1 rs4674344* SNP, A/L ratio, and MetS are significantly associated and T-carriers might have a higher risk of developing MetS due to low A/L ratios in the preclinical stage.

## 1. Introduction

Metabolic syndrome (MetS), a collection of cardiometabolic risk factors that includes obesity, insulin resistance, hypertension, and dyslipidemia, confers increased risks of coronary heart disease and cardiovascular disease (CVD) [1–5]. In terms of mechanism, both adipocytokine and vitamin D are highly associated with MetS.

Among the adipocytokines, the relationships between adiponectin and leptin with MetS are the best established ones. A low adiponectin level has been reported to be correlated with obesity, MetS [6], and CVD [7–13], while a high leptin level has been indicated to be associated with CVD that has close relation to obesity [6, 14–16]. To be integrated, adiponectin/leptin ratio (A/L ratio) has been identified to be highly associated with insulin resistance, MetS, and CVD and even is able to predict CV outcome [17–21].

In addition, vitamin D deficiency has also been reported to be highly associated with MetS [6, 22–25] and to increase the risk of developing CVD, including hypertension, heart failure, and ischemic heart disease [26]. Vitamin D_3_ has been found to be highly associated with adiponectin [27–29], while leptin has been reported to suppress the synthesis of renal 1,25(OH)_2_D_3_ [30]. In the formation of an active form of vitamin D_3_, 1,25(OH)_2_D_3_, two key enzymes of the cytochrome P450 superfamily, sterol 27-hydoxylase (*CYP27A1*) in the liver and 25-hydroxyvitamin D3 1-alpha-hydroxylase (*CYP27B1*) in the kidney, are needed [31].

Vitamin D_3_ and adipocytokine interact with each other and influence the development of MetS. However, in the view of genetics, little about these two vitamin D_3_ metabolic genes with adipocytokines and MetS is studied. Therefore, our purpose was to explore the potential associations of vitamin D_3_ metabolism genes,* CYP27A1* and* CYP27B1*, with adiponectin and leptin and, subsequently, with MetS.

## 2. Material and Methods

### 2.1. Study Population and Protocol

In this cross-sectional study, 694 Taiwanese males (age: 44–77 years) living in Kaohsiung City were recruited from a free health screening program held by our institution [1, 32]. Each of them participated with informed written consent. Ethical approval following the Declaration of Helsinki was authorized by the Institutional Research Ethics Committee of Kaohsiung Medical University Hospital.

**Figure 1:**
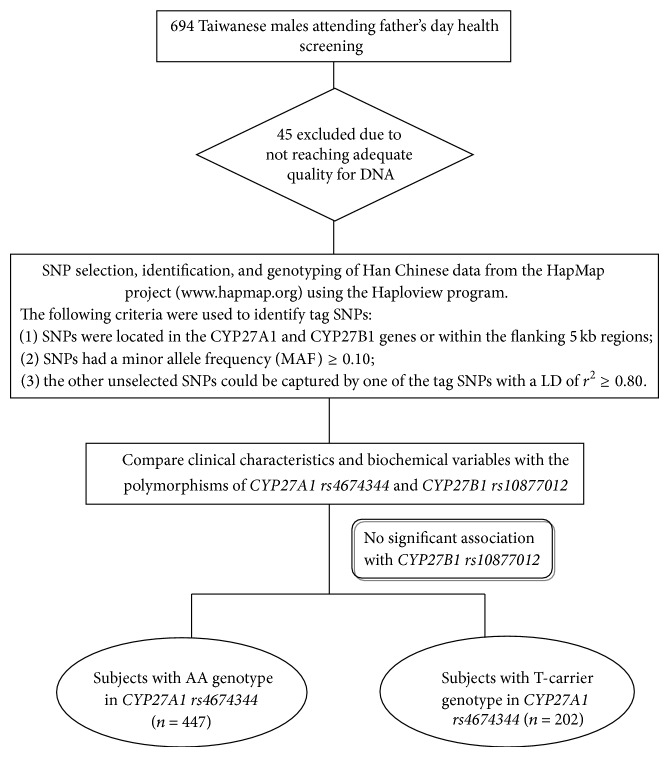
Flow chart of this cohort study.

The men who had a previous diagnosis of hypertension, diabetes mellitus (DM), or hyperlipidemia controlled by regular medication were included in this study, but those who were diagnosed with labile hypertension, labile DM, and advanced liver and/or renal disease were excluded. Entire records of medical, surgical, and psychosexual history, as well as the results of physical examinations including measurements of body weight, height, and blood pressure for each participant, were collected (Figure 1).

The subjects were classified as alcohol drinkers, cigarette smokers, or betel nut chewers if they had regularly consumed any alcoholic beverage ≥1 time per week, smoked ≥10 cigarettes per week, or chewed ≥7 betel quids per week for at least the last 6 months [33, 34]. Hypertension was defined as a systolic blood pressure of ≥140 mmHg or diastolic blood pressure of ≥90 mmHg. Hyperlipidemia was defined as a total cholesterol level of ≥200 mg/dL or triglyceride level of ≥200 mg/dL [1, 35]. DM was diagnosed when the fasting blood glucose (FBG) was ≥126 mg/dL.

### 2.2. Diagnosis of Metabolic Syndrome (MetS)

In accordance with the modified criteria proposed by the Bureau of Health Promotion in Taiwan, MetS was diagnosed once meeting at least three of the following criteria: (1) waist circumference (WC) ≥90 cm; (2) triglycerides (TG) ≥150 mg/dL; (3) high density lipoprotein (HDL) cholesterol <40 mg/dL; (4) blood pressure (BP) ≥130/85 mmHg or current use of antihypertensive medications; (5) FBG >100 mg/dL, previously diagnosed as DM, or current use of oral hypoglycemic agents or insulin [1, 5, 36].

### 2.3. Biochemical Analysis Using Radioimmunoassay

For further biochemical analysis and hormone profiling, each peripheral venous blood sample was drawn after more than 8 hours of fasting overnight into pyrogen-free tube. DiaSorin radioimmunoassay kits (Northwestern Avenue Stillwater, USA; intra-assay CV: 6.8%~11.3%; interassay CV: 12.3%~15.3%) were used to determine the expression of 1,25(OH)_2_D_3_. Millipore radioimmunoassay kits (Missouri, USA) were used to measure the leptin and adiponectin (intra-assay CV: 3.4%~8.3% and 1.78%~6.21%, resp.; interassay CV: 3.0%~6.2% and 6.90%~9.25%, resp.).

### 2.4. Selection, Identification, and Genotyping of the Single Nucleotide Polymorphism

Tag single nucleotide polymorphisms (SNPs) were selected by searching data on Han Chinese in the HapMap project (http://www.hapmap.org/) using Haploview software [37]. Afterward the tag SNPs were identified by the following criteria: (1) SNPs located in the CYP27A1 and CYP27B1 genes or within the flanking 5 kb regions; (2) a minor allele frequency of ≥0.10; and (3) other unselected SNPs that could be captured by one of the tag SNPs with a linkage disequilibrium of *r* ^2^ ≥ 0.80. Selection of SNPs had been completed in March 2006, and only 1 tag SNP had been identified for each of the CYP27A1 and CYP27B1 genes, namely, rs4675344 on the intron 1 region and rs10877012 on the promoter region (Figure 1).

DNA was extracted from the peripheral whole blood using a Puregene DNA Isolation Kit (Gentra Systems Inc., Minneapolis, MN). The two SNPs were determined using a TaqMan 5′ allelic discrimination assay performed on an assays-on-demand SNP genotyping kit (Applied Biosystems, Foster City, CA). SNP amplification assays including 10 ng of sample DNA in 25 *μ*L of reaction solution contained in 12.5 *μ*L of 2x TaqMan Universal PCR Mix (Applied Biosystems) and 1.25 *μ*L of predeveloped assay reagent from the SNP genotyping product (Applied Biosystems) contained in two primers and two MGB-TaqMan probes were performed according to the manufacturer's instructions. Reaction conditions consisted of preincubation at 50°C for 2 min and 95°C for 10 min, followed by 40 cycles at 92°C for 15 s and 60°C for 1 min. Amplifications were performed in an ABI Prism 7500 Sequence Detection System (Applied Biosystems). Our genotyping results were also validated in accordance with the identical results from the 25 patients and 25 controls (about 10% of our participants) who were randomly selected for regenotyping by direct sequencing.

### 2.5. Statistical Analysis

The demographic and laboratory data are presented as mean ± standard deviation (SD). For the controls, the Hardy-Weinberg equilibrium was checked using the chi-square test. The categorical variables and continuous variables were examined by the contingency tables (Pearson chi-square test) and the Student's *t-*test, respectively. Linkage disequilibrium between the polymorphisms was assessed via Haploview software [37]. The ANCOVA test was used to evaluate the associations between the* CYP27A1 rs4675344* and adiponectin/leptin ratio (A/L ratio) controlling for other covariates. Multiple linear regression analysis was used to examine the associations between the A/L ratio and MetS score for the* CYP27A1 rs4675344*. Since the established cardiovascular risk factors including hypertension and DM are the components of MetS, we were only adjusted for sex, age, coronary artery disease (CAD), stroke, alcohol consumption, and smoking status. We also studied the effects of the individual MetS component. All statistical analyses were performed by SPSS 18.0 software (Chicago, IL, USA) with significance levels of *α* = 0.05.

## 3. Results

### 3.1. Patient Characteristics

The baseline characteristics and biochemical data of our subjects with respect to* CYP27A1 rs4675344* were summarized in Table 1. A total of 649 patients had adequate qualities for analysis, of whom 447 (68.9%), 179 (27.6%), and 23 (3.5%) patients had the AA, AT, and TT genotypes, respectively. Due to the low minor allele frequency, the patients with TT and patients with AT were united into the T-carriers for analysis. Compared with the patients with the AA genotype, the T-carriers had significantly higher WC (87.1 ± 6.9 versus 85.7 ± 7.2 cm, *P* = 0.003), positive history of dyslipidemia, total and HDL cholesterol (4.3 ± 1.0 versus 4.1 ± 1.0, *P* = 0.006), leptin (6.5 ± 28.2 versus 4.0 ± 3.3, *P* = 0.005), and A/L ratio (3.7 ± 4.0 versus 5.1 ± 6.0, *P* = 0.001). The T-carriers showed nominal significant number of MetS components (2.1 ± 1.4 versus 1.9 ± 1.4, *P* = 0.04), lower HDL (46.5 ± 11.3 versus 48.5 ± 11.0 mg/dL, *P* = 0.04), adiponectin (10.9 ± 6.2 versus 12.1 ± 7.2 ng/mL, *P* = 0.03), and increased number of MetS components and greater percentages of each MetS component were also noted in the T-carriers compared to those with the AA genotype (Table 2). However, no significant associations were found in the* CYP27B1 rs10877012* SNP.

### 3.2. Regression Analysis of the* CYP27A1 rs4675344* SNP against Other Study Parameters for A/L Ratio

Four models were established for our regression analysis, namely, Model 1 that was adjusted for baseline characteristics including age, BMI, WC, hip circumference, systolic blood pressure, and diastolic blood pressure; Model 2 that was then adjusted for exsmoker, current smoker, ex-betel nut chewer, current betel nut chewer, exdrinker, and current drinker; Model 3 that was further adjusted for comorbidities including DM, hypertension, dyslipidemia, CAD, and stroke; and Model 4 that was farther adjusted for biochemistry data including fasting glucose, total cholesterol, low density lipoprotein (LDL), HDL, and triglycerides (TG). Negative associations between the* CYP27A1 rs4675344 *SNP and the A/L ratio were found consistently from Model 1 to Model 4. In addition, obesity and the parameters related to MetS, including BMI and WC, were also negatively related to the A/L ratio. In Model 4, LDL and fasting glucose were also significantly, negatively associated with the A/L ratio (Table 3).

### 3.3. Associations between the Components of the MetS and* CYP27A1 rs4674344* for the A/L Ratio

**Figure 2:**
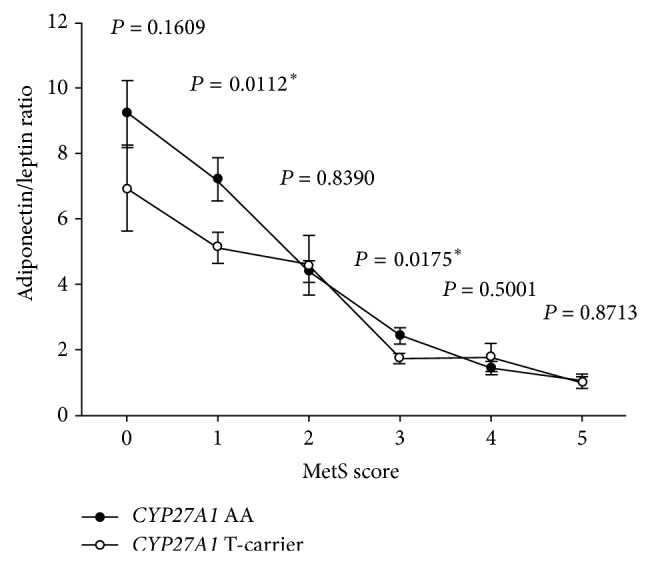
Associations of the adiponectin/leptin ratio with the metabolic syndrome (MetS) scores. The association between adiponectin/leptin ratio and the* CYP27A1 rs4674344 *SNP was more significant in the group with low MetS score than in the group with high MetS score.

**Figure 3:**
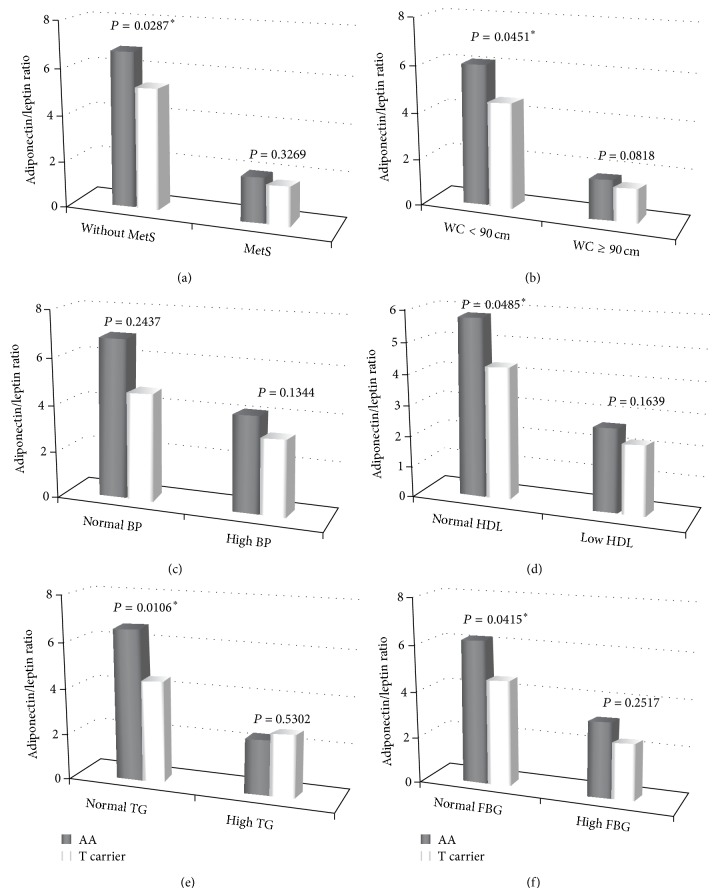
Associations between the* CYP27A1 rs4674344 *SNP and each component of the metabolic syndrome (MetS) for the adiponectin/leptin (A/L) ratio. The A/L ratio was only significant as the condition did not meet all individual MetS criteria except for the BP component after adjusting for age, BMI, smoking, alcohol drinking, betel nut chewing, CAD, total cholesterol, LDL, CHOL(T)/HDL, and the other 4 components, respectively, for the* CYP27A1 rs4674344 *SNP. BMI: body mass index; BP: blood pressure; DM: diabetes mellitus; CHOL(T): total cholesterol; HDL: high density lipoprotein; TG: triglycerides.  ^*^Significant difference (*P* < 0.05).

The A/L ratio showed greatest association with the* CYP27A1 rs4674344 *SNP, and the MetS also had significant association, too. Therefore, we further examined the influence of MetS and its components on the associations between the* CYP27A1 rs4674344 *SNP and A/L ratio. Greater association between A/L ratio and the* CYP27A1 rs4674344 *SNP was noted in the low MetS score group when compared to the high MetS score group (Figure 2). Significant A/L ratio was only found when most of the MetS components were in the preclinical stage (not meeting individual criteria). However, there was no significant association with the BP component after adjusting for age, BMI, smoking, alcohol drinking, betel nut chewing, CAD, total cholesterol, LDL, HDL, and the other 4 components for the* CYP27A1 rs4674344 *SNP (Table 4, Figure 3).

## 4. Discussion

This study selected the SNPs by searching Han Chinese data from the HapMap project (http://www.hapmap.org/) using Haploview software [37]. These SNPs were located in the CYP27A1 genes and CYP27B1 genes or within the flanking 5 kb regions with a minor allele frequency ≥0.10. Accordingly, the other unselected SNPs could be captured by one of the tag SNPs with a linkage disequilibrium of *r* ^2^ ≥ 0.80. As a result, only 1 tag SNP was obtained for each of the CYP27A1 and CYP27B1 genes, namely, the rs4675344 on intron 1 region and the rs10877012 on the promoter region (Figure 1).

The MetS and its HDL component are significantly associated with the* CYP27A1 rs4674344 *SNP. CYP27A1 mRNA expression is also significantly associated with MetS in one in vivo study [38]. The HDL cholesterol has been reported to have a strong correlation with the adiponectin and to affect the adipocyte metabolism and adiponectin expression [39, 40]. And it is known that mutations in CYP27A1 are associated with cerebrotendinous xanthomatosis, a rare lipid storage disease with the deposition of a form of cholesterol (cholestanol) in the brain and other tissues [41–44]. Our study provides further novel information of the impact of* CYP27A1 rs4674344 *SNP on both of them. However, like previous document, no CYP27 SNPs in our study were associated with 1,25(OH)(2)D levels [45].

A/L ratio is the best surrogate for inflammatory burden and insulin resistance [46, 47]. Adiponectin acts as an insulin-sensitizing adipocytokine and an anti-inflammatory factor especially with regard to atherosclerosis [48]. Leptin is known to be a satiety factor regulating body weight by suppressing appetite and stimulating energy expenditure. Leptin is also a proinflammatory adipocytokine, which induces the development of T helper 1 cells that contribute to the progress of inflammation [49–51]. For in-depth analysis in our study, A/L ratio was not only significantly higher in the* CYP27A1 rs4674344 *SNP T-carriers than in those with the AA genotype (3.7 ± 4.0 versus 5.1 ± 6.0, *P* = 0.001), but was also most correlated with this SNP. Against other study parameters for the A/L ratio, the* CYP27A1 rs4675344* SNP consistently had significantly negative association with the A/L ratio from Model 1 to Model 4 after adjusting for baseline characteristics, life habits, comorbidities, and biochemistry profiles. In addition, obesity and MetS-related parameters including BMI, WC, and hip circumference were also negatively associated with the A/L ratio. In Model 4, LDL and fasting glucose were significantly negatively associated with the A/L ratio, too (Table 3). These results all indicated that the* CYP27A1 rs4675344* SNP, A/L ratio, and MetS are closely associated with each other.

Another set of novel in-depth information provided by our study related to the* CYP27A1 rs4674344 *SNP was that the A/L ratio was only significantly different in the preclinical stage of most individual MetS criteria, except for the BP component, but not in the clinical stage of MetS. The SNPs of genes are imprinted in the germ-cell stage and one previous investigation had also reported that the changes of A/L ratio and development of the MetS partly accorded with one's own genetics [52]. Therefore, our novel findings indicated that those T-carriers with the* CYP27A1 rs4674344 *SNP tend to have a higher risk of developing MetS, owing to an adipocytokine imbalance. However, the* CYP27A1 rs4674344 *SNP did not seem to play a role in the development of BP component.

Based on our findings and the previous evidence, the* CYP27A1 rs4674344* might participate in lipid and adipocytokine homeostasis and in the crosstalk of HDL with adiponectin. However, unequal number of subjects with the polymorphism of* CYP27A1 rs4674344* was the main limitation in this study, which might lead to bias in the results. A large-scale cohort study with equal numbers of subjects may be dedicated to investigate the refined causality, including the SNP of* CYP27A1 rs4674344* for the major cardiovascular events.* In vitro* studies may also be needed to clarify the comprehensive role of the* CYP27A1 rs4674344* SNP.

## 5. Conclusions

This study reported the novel finding that vitamin D_3_ metabolism gene* CYP27A1 rs4674344* was closely associated with A/L ratio and MetS. The A/L ratio alone had significant association with the* CYP27A1 rs4675344* SNP. In addition, for the* CYP27A1 rs4674344 *SNP, the A/L ratio was only significantly different in the preclinical stage of the most individual MetS criteria except the BP component. This finding indicated that the T-carriers were at a higher risk of developing MetS.

## Figures and Tables

**Table 1:** Means ± standard deviations or numbers (%) of the baseline characteristics and biochemical variables in the patients with the *CYP27A1 rs4674344* SNP.

	Subjects with AA (*n* = 447)	Subjects with T-carrier (*n* = 202)	*P* value
Age (yr)	55.5 ± 4.1	56.1 ± 5.1	0.11
BMI (kg/m^2^)	25.6 ± 7.1	25.6 ± 2.9	0.93
Waist circumference (cm)	85.7 ± 7.2	87.1 ± 6.9	0.03^*^
Hip circumference (cm)	100.4 ± 64.5	97.8 ± 5.5	0.57
SBP (mm-Hg)	130.9 ± 12.4	130.5 ± 11.1	0.75
DBP (mm-Hg)	82.1 ± 8.6	82.3 ± 8.7	0.86
DM, *n* (%)	4.2 (9.4)	27 (13.4)	0.14
Hypertension, *n* (%)	124 (27.7)	66 (32.7)	0.20
Smoking, *n* (%)			0.36
Nonsmokers	331 (74.0)	140 (69.3)	
Former smokers	62 (13.9)	30 (14.9)	
Current smokers	54 (12.1)	32 (15.8)	
Dyslipidemia history, *n* (%)	76 (16.8)	53 (26.2)	0.006^*^
CAD history, *n* (%)	30 (6.7)	15 (7.4)	0.76
Stroke history, *n* (%)	3 (0.7)	5 (2.5)	0.056
Betel quid, *n* (%)	2 (0.4)	1 (0.5)	0.87

Blood biochemistry			
Fasting glucose (mg/dL)	99.6 ± 20.6	101.2 ± 22.2	0.36
Triglycerides (mg/L)	134.1 ± 98.6	137.7 ± 88.8	0.66
Total cholesterol (mg/dl)	188.6 ± 33.4	191.9 ± 35.8	0.25
HDL-C (mg/dL)	48.5 ± 11.0	46.5 ± 11.3	0.04^*^
CHOL(T)/HDL	4.1 ± 1.0	4.3 ± 1.0	0.006^*^
Adiponectin (ng/mL)	12.1 ± 7.2	10.9 ± 6.2	0.03^*^
Leptin (ng/mL)	4.0 ± 3.3	6.5 ± 28.2	0.005^*^
Adiponectin/leptin	5.1 ± 6.0	3.7 ± 4.0	0.001^*^
1,25(OH)_2_D_3 _(pg/mL)	45.6 ± 19.0	43.3 ± 17.2	0.14

BMI: body mass index; SBP: systolic blood pressure; DBP: diastolic blood pressure; DM: diabetes mellitus; CAD: coronary artery disease; CHOL(T): total cholesterol; HDL: high density lipoprotein. ^*^Significant difference (*P* < 0.05).

**Table 2:** Means ± standard deviations or numbers (%) of the MetS parameters in the patients with the *CYP27A1 rs4674344* SNP.

	Subjects with AA (*n* = 447)	Subjects with T-carrier (*n* = 202)	*P* value of the association with *CYP27A1 rs4674344 *
MetS, *n* (%)			0.038^*^
Yes	158 (35.4)	90 (44.6)	
No	289 (64.6)	112 (55.4)	
Component numbers:			
0 (*n* = 99)	78 (17.8)	21 (10.4)	
1 (*n* = 171)	113 (25.5)	58 (28.7)	
2 (*n* = 129)	96 (21.6)	33 (16.3)	
3 (*n* = 142)	90 (20.2)	52 (25.7)	
4 (*n* = 83)	53 (11.9)	30 (14.9)	
5 (*n* = 23)	15 (3.3)	8 (4.0)	
Number of MetS criteria	1.9 ± 1.4	2.1 ± 1.4	0.038^*^
Individual MetS Component, *n* (%)			
High waist circumference	118 (26.3)	65 (32.1)	0.26
High blood pressure	273 (61.1)	127 (62.9)	0.38
Low HDL	95 (21.2)	55 (27.2)	0.25
High triglyceride	150 (33.6)	85 (42.1)	0.06
High fasting glucose	160 (35.8)	79 (39.1)	0.51

MetS: metabolic syndrome; HDL: high density lipoprotein. *P* values were adjusted for age, alcohol, smoking, and betel chewing. ^*^Significant difference (*P* < 0.05).

**Table 3:** *β*-coefficients between the *CYP27A1 rs4674344* SNP and the adiponectin/leptin ratio.

	Model 1		Model 2		Model 3		Model 4	
	*β*-coefficient	*P* value	*β*-coefficient	*P* value	Β-coefficient	*P* value	*β*-coefficient	*P* value
*CYP27A1 rs4674344 *	−0.58	0.0092^*^	−0.6	0.0077^*^	−0.57	0.0158^*^	−0.51	0.0257^*^
Age	0.03	0.6655	0.03	0.6287	0.05	0.4556	0.06	0.3401
BMI	−0.51	0.0001^*^	−0.51	0.0002^*^	−0.51	0.0005^*^	−0.36	0.0108^*^
WC	−0.39	<0.0001^*^	−0.38	<0.0001^*^	−0.38	<0.0001^*^	−0.3	<0.0001^*^
HC	0.23	0.0006^*^	0.22	0.0010^*^	0.21	0.0026^*^	0.14	0.0493^*^
SBP	−0.08	0.0513	−0.08	0.0399	−0.08	0.0638	−0.05	0.1762
DBP	0.02	0.4605	0.02	0.5053	0.02	0.4651	0.02	0.4403
Exsmoker			−0.54	0.2496	−0.44	0.3763	−0.33	0.4908
Current smoker			0.31	0.4731	0.18	0.6967	0.15	0.729
Ex-betel nut chewer			−0.02	0.9961	−0.21	0.9507	−0.16	0.9597
Current betel nut chewer			−0.05	0.9776	0.22	0.907	0.34	0.853
Exdrinker			−0.28	0.6513	−0.58	0.3906	−0.63	0.3326
Current drinker			0.96	0.3427	1.39	0.2231	1.35	0.2198
DM					−0.17	0.6799	0.21	0.6329
Hypertension					−0.09	0.7559	0.02	0.9296
Dyslipidemia					−0.5	0.0887	−0.14	0.6479
CAD					−0.53	0.2821	−0.34	0.4727
Stroke					−2.27	0.3797	−2.12	0.3933
Fasting glucose							−7.02	0.0414^*^
CHOL(T)							14.3	0.2066
LDL							−13.04	0.0125^*^
HDL							4.25	0.5984
TG							−7.73	0.0003^*^

Model 1 is adjusted for baseline characteristics, including age, BMI (body mass index), WC (waist circumference), HC (hip circumference), SBP (systolic blood pressure), and DBP (diastolic blood pressure); Model 2 is further adjusted for habits, including being exsmoker, current smoker, ex-betel nut chewer, current betel nut chewer, exdrinker, and current drinker; Model 3 is further adjusted for comorbidities, including DM (diabetes mellitus), hypertension, dyslipidemia, CAD (coronary artery disease), and stroke; Model 4 is further adjusted for biochemistry data, including fasting glucose, CHOL(T) ( total cholesterol), LDL (low density lipoprotein), HDL (high density lipoprotein), and TG (triglycerides). ^*^Significant difference (*P* < 0.05).

**Table 4:** Associations between the *CYP27A1 rs4674344 *SNP and each component of the metabolic syndrome for the adiponectin/leptin ratio.

		MetS		WC 90 cm		High BP		Low HDL		High TG		High FBG	
		No	Yes	No	Yes	No	Yes	No	Yes	No	Yes	No	Yes
	*n*	401	248	451	198	215	434	487	162	396	253	393	256
A/L ratio	AA genotype	6.80 ± 0.40	1.97 ± 0.16	6.16 ± 0.36	1.70 ± 0.14	6.92 ± 0.60	4.14 ± 0.29	5.83 ± 0.35	2.70 ± 0.36	6.65 ± 0.42	2.42 ± 0.16	6.26 ± 0.42	3.22 ± 0.27
	T-carrier	5.28 ± 0.43	1.68 ± 0.17	4.55 ± 0.36	1.45 ± 0.13	4.65 ± 0.60	3.28 ± 0.30	4.26 ± 0.35	2.22 ± 0.37	4.47 ± 0.39	2.69 ± 0.38	4.60 ± 0.41	2.39 ± 0.30
	*P* value	0.0287^*^	0.3269	0.0451	0.0818	0.2437	0.1344	0.0485	0.1639	0.0106	0.5302	0.0415	0.2517

The model was adjusted for age, BMI, smoking, alcohol drinking, betel nut chewing, CAD, total cholesterol, LDL, CHOL(T)/HDL, and the other 4 components, respectively, for the *CYP27A1 rs4674344* SNP. A/L ratio: adiponectin/leptin ratio; MetS: metabolic syndrome; WC: waist circumference; BP: blood pressure; HDL: high density lipoprotein; TG: triglycerides; FBG: fasting blood glucose; BMI: body mass index; CHOL(T): total cholesterol. ^*^Significant difference in MetS (*P* < 0.05); significant difference in 5 MetS components (*P* < 0.01).
